# Acute and Subchronic Exposure to Hemp (*Cannabis sativa* L.) Leaf Oil: Impacts on Vital Organs in Sprague-Dawley Rats

**DOI:** 10.3390/ph18101437

**Published:** 2025-09-25

**Authors:** Putcharawipa Maneesai, Monchai Duangjinda, Chanon Labjit, Juthamas Khamseekaew, Prapassorn Potue, Anuson Poasakate, Poungrat Pakdeechote

**Affiliations:** 1 Department of Physiology, Faculty of Medicine, Khon Kaen University, Khon Kaen 40002, Thailand; putcma@kku.ac.th (P.M.); juthakh@kku.ac.th (J.K.); prappo@kku.ac.th (P.P.); 2 Department of Animal Science, Faculty of Agriculture, Khon Kaen University, Khon Kaen 40002, Thailand; monchai@kku.ac.th; 3 Department of Horticulture, Faculty of Agriculture, Khon Kaen University, Khon Kaen 40002, Thailand; lchano@kku.ac.th; 4 Institute of Medicine, Suranaree University of Technology, Nakhon Ratchasima 30000, Thailand; anuson.po@sut.ac.th

**Keywords:** *Cannabis sativa* L., hemp, hemp leaf, vital organs, safety assessment

## Abstract

**Background/Objectives**: Hemp (*Cannabis sativa* L. subsp. *sativa*) is a plant within the *Cannabis sativa* species and utilized for several applications, including antioxidation, antihypertension, and anti-inflammation. To our knowledge, no prior study has assessed the acute and sub-chronic oral safety of hemp leaf oil in Sprague-Dawley rats under Thailand-compliant THC levels. This study investigates the acute and sub-chronic effects of Hemp leaf oil (HLO) on the heart, liver, and kidneys of male and female Sprague-Dawley rats. **Methods**: Six-week-old male and female Sprague-Dawley rats were administered HLO (1.5 mL/kg) intragastrically, either as a single dose or a repeat dose over 28 days. **Results**: No changes in body or organ weights were observed following acute and sub-chronic HLO administration in sex-matched groups. Moreover, blood pressure and heart rate remained comparable across groups after acute and sub-chronic HLO treatment. Both acute and sub-chronic administration of HLO did not influence electrolyte balance, liver enzymes, total protein, albumin, blood urea nitrogen, or creatinine levels. Hematoxylin and eosin staining revealed the normal morphology of the heart, liver, and kidneys in rats subjected to HLO, during both acute and sub-chronic treatment. **Conclusions**: In conclusion, our data suggested that both acute and sub-chronic administration of HLO at 1.5 mL/kg could be safe for the vital organs. These findings support the potential use of HLO in therapeutic applications, particularly in scenarios when the safety of essential organs is at stake.

## 1. Introduction

Hemp (*Cannabis sativa* L. subsp. *sativa*) is an industrial plant widely used in textiles, pulp and paper, cosmeceuticals, pharmaceuticals, and herbal medicine [1,2]. Hemp extracts can be taken from any part of the hemp plant. The yield of bioactive compounds from hemp extract provides many beneficial effects for health, such as antidepressant, anti-inflammatory, antioxidant, antiproliferative properties, and hypoglycemic effects [3,4]. Among the bioactive compounds in the hemp plant, the psychoactive tetrahydrocannabinol (THC) and the non-psychoactive cannabidiol (CBD) have gained more attention in research due to their potential medicinal benefits [5,6]. The extracts from hemp plants grown in Thailand, which contain less than 0.2% THC, are now legally permitted according to the Ministry of Public Health’s Notification (Re: Prescribing the List of Category 5 Narcotics, B.E. 2565/2022) under the Narcotics Code [7].

Various parts of the hemp plant have been widely utilized as valuable sources of nutraceuticals and functional foods, such as hemp seed, hemp seed oil, and hemp inflorescence. Nutrient-rich hemp seeds and their derivatives, such as oil and protein extracts, with very low THC content [8], are considered a plant-based option within the hemp food industry. Previous studies have reported that dietary hemp seeds are more effective than their extracted oil in attenuating metabolic disorders in obese rats, due to their high nutrient and bioactive compound content [9], including terpenes, flavonoids, phytocannabinoids, phenolic acids, and carotenoids [8,9]. The inflorescence and leaves of the hemp plant are also used as sources of phytocannabinoids. However, they differ in THC concentration, with the inflorescence having more (0.24–0.47%) than the leaf (0.03–0.06%) [10].

Hemp leaves, which are considered byproducts of industrial hemp [11], have been found to contain higher phytochemical concentrations compared to other parts of the plant [12] and have been reported to be used in traditional medicine. Therefore, industrial hemp leaves could be a good source for extraction and product development for medical purposes. A recent study using exhaustive swimming tests in mice demonstrated that the water extract of hemp leaves reduced the accumulation of lactic acid and improved the activity of antioxidant defense enzymes, indicating its anti-fatigue properties [13]. Chatzimitakos et al. found anti-inflammatory activity in a water extract of hemp leaves, which can be increased under specific extraction conditions [14]. Similarly to other botanical extracts, many factors, such as hemp variety, cultivation methods, and extraction techniques may influence the phytochemical content of hemp extracts. For example, decreasing hemp extraction duration and augmenting the liquid-to-solid ratio enhanced efficacy and maximized polyphenol recovery [14]. These factors may impact the bioactivity and toxicological risks of the extracts. Our prior investigation evaluated the antihypertensive efficacy of *Cannabis sativa* L. Leaf Oil (1 mL/kg as a therapeutic dose) in hypertensive rats [15]. Therefore, safety assessments are essential before public use. In this study, RPF3 hemp leaves from the variety grown in Thailand were extracted using a two-step solvent extraction process. The resulting extracts were then used to evaluate their impact, focusing on general appearance, blood pressure, biochemical analysis, and histopathology of vital organs in both an acute oral administration study and a 28-day repeat-dose oral administration study in Sprague-Dawley rats.

## 2. Results

### 2.1. Effects of HLO on Mortality, General Appearance, and Neurobehavior of Sprague-Dawley Rats

Both the acute and the 28-day repeat-dose oral administration study showed no mortality following the administration of HLO or vehicle (olive oil). In addition, no alterations in general appearance or neurobehavior, were observed in the HLO-treated groups throughout the experimental period.

### 2.2. Effects of HLO on Body Weight, Food Intake and Water Consumption in Sprague-Dawley Rats

**Figure 1:**
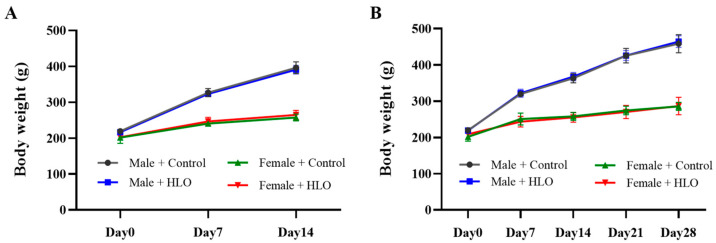
Weekly body weight gain for the acute oral administration study (**A**) and 28-day repeat-dose oral administration study (**B**) in the Sprague-Dawley rats. Data are presented as mean ± standard deviation (n = 5/group). HLO, hemp leaf oil.

The effects of HLO on average body weight gain during both the acute and the 28-day repeat-dose oral administration study are presented in Figure 1. The results show no significant differences in mean weekly body weight gain between the HLO-treated group and their sex-matched controls, with both sexes exhibiting normal growth patterns in Sprague-Dawley rats. Additionally, food and water consumption did not differ significantly between the HLO-treated group and their sex-matched controls (Table 1).

### 2.3. Effects of HLO on the Vital Organ Weight of Sprague-Dawley Rats

Both the acute and the 28-day repeat-dose oral administration study showed no significant differences in the weights of vital organs, including heart, liver, and kidneys, between the HLO-treated group and their sex-matched controls (Table 2).

### 2.4. Effects of HLO on Blood Pressure and Heart Rate of Sprague-Dawley Rats

**Figure 2:**
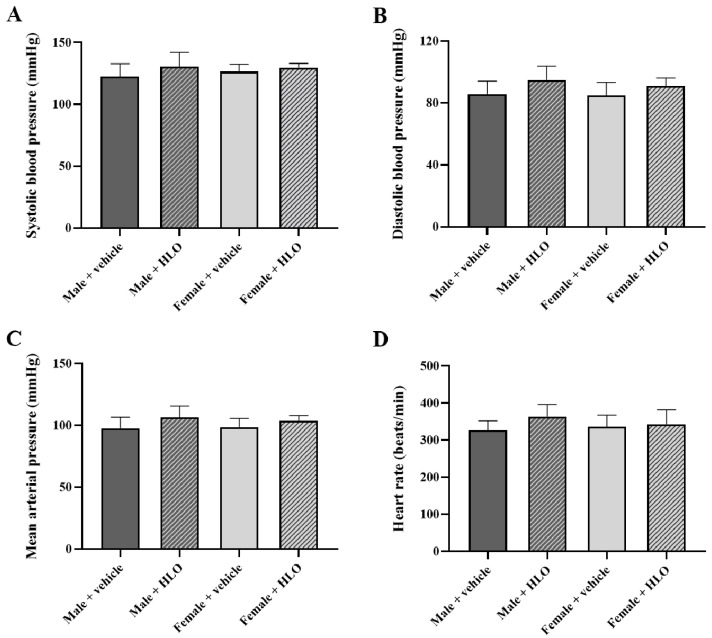
Blood pressure parameters of acute HLO treatment, systolic blood pressure (**A**), diastolic blood pressure (**B**), mean arterial pressure (**C**), and heart rate (**D**) measured by the tail-cuff method for the acute oral administration study in Sprague-Dawley rats at the end-point of the study. Data are presented as mean ± standard deviation (n = 5/group). Statistical analysis in each sex was evaluated by unpaired *t*-test. HLO, hemp leaf oil.

**Figure 3:**
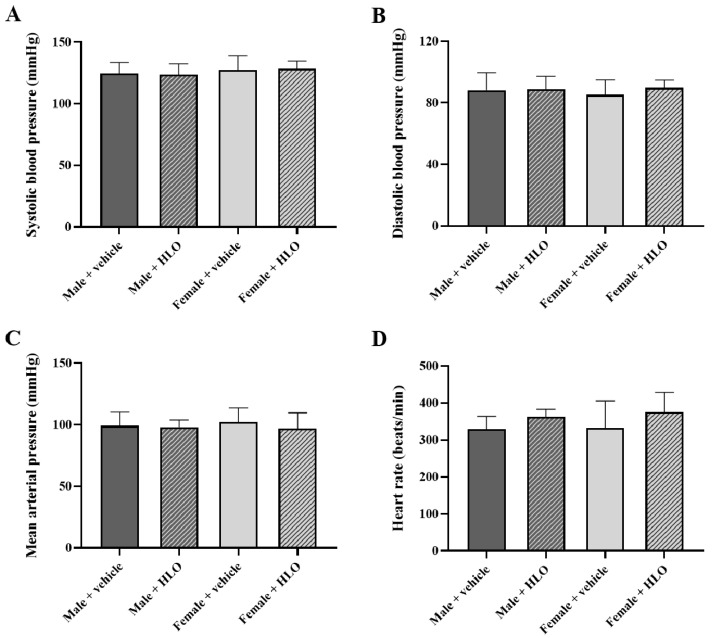
Blood pressure parameters of sub-chronic HLO treatment, systolic blood pressure (**A**), diastolic blood pressure (**B**), mean arterial pressure (**C**), and heart rate (**D**), were measured using the tail-cuff method for the 28-day repeat-dose oral administration study in Sprague-Dawley rats at the study endpoint. Data are presented as mean ± standard deviation (n = 5/group). Statistical analysis in each sex was evaluated by an unpaired *t*-test. HLO, hemp leaf oil.

Administration of HLO did not produce any significant changes in systolic blood pressure, diastolic blood pressure, mean arterial pressure, or heart rate in both the acute (Figure 2) and the 28-day repeat-dose oral administration study (Figure 3).

### 2.5. Effects of HLO on Biochemical Parameters of Sprague-Dawley Rats

The clinical biochemical parameters measured at the termination of both the acute and the 28-day repeat-dose oral administration study in Sprague-Dawley rats are shown in Table 3 and Table 4, respectively. All parameters from the control and HLO-treated groups of both sexes remained within the normal range for each sex. These findings suggest that the administration of HLO during the experimental time course did not produce any biologically or toxicologically significant effects.

### 2.6. Effects of HLO on Macroscopic Lesions and Histopathology of Sprague-Dawley Rats

**Figure 4:**
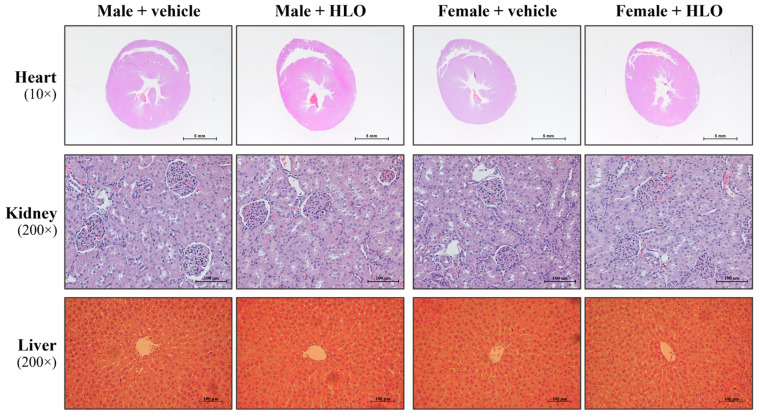
Representative image of hematoxylin and eosin (H&E) staining of the ventricular cross-sectional morphology, kidney tissue section, and liver tissue section from the 28-day repeat-dose oral administration study in Sprague-Dawley rats, observed under a light microscope (image magnification and scale bar as indicated). HLO, hemp leaf oil.

No macroscopic pathological lesions were observed in either the acute or the 28-day repeat-dose oral administration study following HLO or olive oil administration. Histopathological examination of selected organs from the 28-day repeat-dose oral administration study in male and female Sprague-Dawley rats showed normal tissue structures in all groups. Representative histological images of the heart, kidney, and liver are shown in Figure 4.

## 3. Discussion

This study determined that the phytochemical composition of HLO includes 0.40 mg/g CBD and less than 0.02 mg/g THC, which complies with Thailand FDA requirements for food and beverage products [7]. Microbiological analysis of HLO samples revealed no contamination by foodborne pathogens, including *Escherichia coli*, *Salmonella* spp., and *Staphylococcus aureus*. Furthermore, testing for heavy metals, including arsenic, cadmium, lead, mercury, and tin, was under permissible limits, signifying that the HLO sample was free from foodborne microorganisms and heavy metals, thus rendering it safe for consumption.

We discovered that male and female Sprague-Dawley rats that received a single oral dosage or a 28-day repeat dose of HLO (1.5 mL/kg/day) exhibited no mortality, alterations in general appearance, or neurobehavioral abnormalities. There was no significant difference in body weight growth following 14 days of single oral treatment of HLO (1.5 mL/kg) in male and female rats. Similarly, a 28-day repeat dose of HLO did not affect body weight or weight gain in sex-matched rats. The findings indicated that neither the acute nor sub-chronic effects of HLO impacted rat body weight. These findings could imply that the metabolic rate or energy expenditure of rats remained unchanged [16,17] and that all rats maintained good health throughout the study. The results corroborated this hypothesis, as no variation in food intake or water consumption was detected across all groups of rats. Consequently, acute and sub-chronic treatment of HLO did not induce toxicity in major organs, as the heart, liver, or kidney weights were comparable to those of control rats in the same sex groups. Our findings align with a prior study indicating that subchronic oral toxicity assessments of elixinol hemp extract in rats do not influence body weight or organ weight, except for an increase in liver weight and hepatocellular hypertrophy at elevated doses; however, there were no alterations in clinical chemistry to suggest liver damage [18]. For both male and female Sprague-Dawley rats, acute and sub-chronic administration of hemp extract in an olive oil matrix did not result in any discernible negative effects or significant systemic toxicity [19].

This study demonstrated the safety impact of HLO on essential organs, including the heart, kidneys, and liver. The cardiovascular effects of HLO were validated, since both acute and sub-chronic administration of HLO did not alter blood pressure or heart rate in sex-matched cohorts. It is well known that THC, a principal active component of cannabis, is recognized for its neurobehavioral and cardiovascular effects [20,21,22]. Nonetheless, HLO administration did not elicit neurobehavioral impairment, hypotension, hypertension, bradycardia, or tachycardia in this investigation due to its extremely low THC concentration. A recent publication corroborated our findings that acute treatment with hemp extract did not impact behavioral or physiological changes, systolic blood pressure, diastolic blood pressure, mean arterial blood pressure, or heart rate in male and female rats [23]. The acute and sub-chronic administration of HLO had no effect on electrolyte balance in rats. These results imply that renal and endocrine functions could be normal [24]. The safety impact of HLO on renal function was validated by the levels of BUN and creatinine [25], which showed no significant difference between the vehicle and HLO-treated rat in sex-matched groups. Moreover, acute and sub-chronic HLO administration did not produce changes in liver function and lipid metabolism, as seen by the absence of differences in liver function enzymes, total protein, albumin, total bilirubin and cholesterol in circulation across sex-matched rat groups. Previous studies have reported the beneficial effect of hemp seed oil including improved liver function and lipid metabolism in lean and obese Zucker rats [26,27]. Acute and sub-chronic HLO therapy did not result in aberrant macroscopic lesions or histology in the heart, kidney, or liver. These data indicate that HLO has safety effects on the morphology of the heart, kidney, and liver. Our study demonstrated that HLO is safe for vital organs. Our findings were consistent with those of a prior study, which found that acute and sub-chronic administration with a whole plant ethanol extract of hemp in male and female rats resulted in normal liver enzyme levels, kidney function indicators, and histological assessments of the liver and kidneys [28]. Evidence indicated that sub-chronic administration of hemp seed oil rich in fatty acids did not influence liver enzymes, urea, and creatinine levels in rats [29]. It could suggest that our results in HLO could fill the gap between the safety of bioactive components of different parts of hemp. HLO has beneficial components like terpenes and phenolic compounds, while hemp seed oil has nutraceuticals like fatty acids (including linoleic acid (omega-6), α-linolenic acid (omega-3), and oleic acid), tocopherols, and amino acids [30,31].

A prior investigation demonstrated the cardioprotective properties of HLO in hypertensive rats [15]. Hemp seed oil has been shown to mitigate vascular dysfunction in Zucker rats [32]. Furthermore, hemp seed oil exhibits hepatoprotective properties in fatty liver disease generated by a high-fat diet [33]. However, this study has certain limitations: (1) it did not assess the dose–response relationship of HLO on vital organs. The maximum tolerated dose of HLO was not determined, and the selected dose of 1.5 mL/kg was based on recommendations to prevent mortality or adverse respiratory effects. We acknowledge the importance of a dose–response evaluation in conventional toxicology. A dose–response evaluation significantly enhances the toxicological characterization of HLO, confirming the calculation of the no observed adverse effect level (NOAEL) and providing an analytical basis for calculating margins of safety and human-equivalent doses. For this study, a single-dose design was selected as our aim was not to determine a toxicity threshold, but to verify the absence of adverse effects at an effective dose (1.5 mL/kg) for reducing blood pressure and cardiovascular complications noted in prior research [1]. Given that no adverse effects were seen on this dosage in animals, our results strongly affirm the product’s safety for its intended use. (2) This work concentrated on acute and sub-chronic evaluations of HLO; nevertheless, chronic assessments aimed at investigating carcinogenicity, reproductive toxicity, and possible bioaccumulation are crucial domains for future research. Our findings offer crucial baseline data that will guide the design and interpretation of further chronic exposure assessments, enhancing the comprehension of HLO’s toxicological profile across various temporal exposure contexts.

Our findings may substantiate product safety standards or risk assessments, as HLO includes THC levels below 0.2%, aligning with regulatory criteria in Thailand, the European Union (e.g., EFSA guidelines) [34], and FDA regulations in the United States [35,36]. The safety impacts on vital organs in both sexes throughout our acute and 28-day sub-chronic study provide evidence that could inform global risk evaluations and establish safety margins for HLO products in clinical applications.

## 4. Materials and Methods

### 4.1. The Preparation Process of Hemp Leaf Oil

The *Cannabis sativa* L. subsp. *sativa* plants were grown from RPF3 hemp variety seeds developed by the Highland Research and Development Institute, Ministry of Agriculture and Cooperatives, Government of Thailand. This hemp was planted with a 14 to 16 h light duration during the vegetative stage and cultivated in experimental plots at the Cannabis Research Farm, Faculty of Agriculture, Khon Kaen University, Thailand (16.471° N, 102.8115° W). Nitrogen fertilization (300 kg/ha) was applied, and the planting density was set at 6000 seedlings per hectare. The hemp leaves were harvested at the early-flowering stage, then chopped into small pieces and dried at 40 °C for 3 to 5 days. After drying, they were stored in zip-lock plastic bags at 25 °C. The hemp extract was prepared using a two-step solvent extraction method. In the first step, 5 kg of the hemp leaves were immersed in 2 L of cellulolytic enzyme solution in a closed container for 7 days to digest the cell wall. The aqueous phase was then mixed with 1.5 L of absolute ethanol at 25 °C. In the second step, the extract was dissolved in a water–ethanol mixture at 80 °C, followed by ethanol evaporation under a 50-mbar vacuum for at least 4 h. Finally, 1 L of the extract was mixed with food-grade olive oil in a 1:4 ratio to produce edible HLO. This final extract contains 0.40 mg/g CBD and less than 0.02 mg/g THC (Table 5), as determined by high-performance liquid chromatography (HPLC) analysis. These values are compliant with Thailand FDA regulations for food and drink products [7].

The specifications of the test article, HLO, used in this study are shown in Table 5. Additionally, the HLO sample was tested for contamination by foodborne pathogens, including *Escherichia coli*, *Salmonella* spp., and *Staphylococcus aureus*, as well as for heavy metals such as As, Cd, Pb, Hg, and Sn. The results showed that the HLO sample was free from foodborne pathogens, and the heavy metals were undetectable (Table 5).

### 4.2. Experimental Animals

The 6-week-old male and female Sprague-Dawley rats purchased from Nomura Siam International Co., Ltd., Bangkok, Thailand, were used in the acute oral administration study and the 28-day repeat dose oral administration study. All animal procedures followed the standards for the care and use of experimental animals and were approved by the Animal Ethics Committee of Khon Kaen University, Khon Kaen, Thailand (IACUC-KKU-11/65). The animals were housed in a heating, ventilation, and air-conditioning (HVAC) system at 23 ± 2 °C and 30–60% relative humidity, with a 12 h light/dark cycle. All rats had free access to food (standard rat chow) and water. After a week of acclimatization, the rats of both sexes were randomly divided into two groups: the control group, which received olive oil as a vehicle, and the testing group, which received HLO at 1.5 mL/kg BW/day. Both the vehicle and HLO were administered to the rats via oral gavage using a stainless steel intragastric needle. This study used a gavage volume of 1.5 mL/kg BW/day to minimize the risk of mortality or unfavorable respiratory effects [44].

### 4.3. Treatments

In the acute oral administration study, twenty male Sprague-Dawley rats (209–227 g) and twenty female Sprague-Dawley rats (179–220 g) were randomly assigned to the treatment groups (n = 5/group/sex). The HLO-treated group received a single oral dose of HLO on Day 0 of the experiment. The dose selection was based on a previous study [15]. Following administration, all animals were monitored for harmful effects or death for 24 h, and subsequently, daily observation for clinical signs of ill health for 14 days.

**Figure 5:**
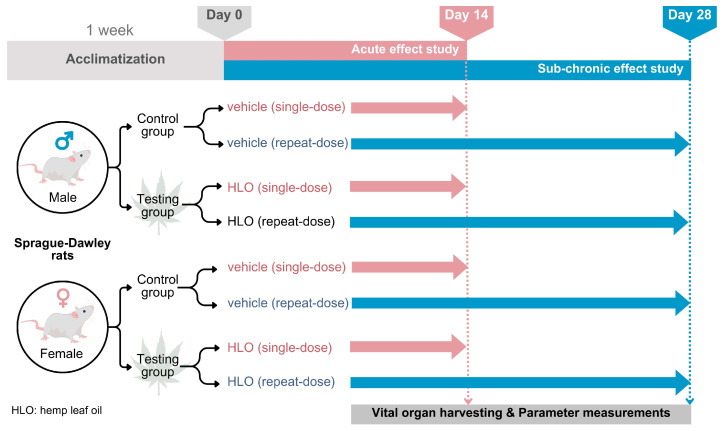
Experimental designs.

In the 28-day repeat-dose oral administration study, twenty male Sprague-Dawley rats (214–223 g) and twenty female Sprague-Dawley rats (187–215 g) were assigned to the treatment groups, stratified by body weight to ensure comparable mean body weight within each sex (n = 5/group/sex). The HLO-treated group in both sexes received HLO at a dose of 1.5 mL/kg BW/day, administered once daily for 28 consecutive days. General observations were conducted daily throughout the treatment period to monitor general health and detect any clinical signs of illness. The experimental designs were summarized as depicted in Figure 5.

### 4.4. General Assessments, Body Weight, Food Intake and Water Consumption Determination

The animal’s body weight was measured weekly to assess the effects of HLO on growth in Sprague-Dawley rats. Food and water intake were recorded daily for each cage, with feed consumption calculated as the amount consumed/rat/day. Clinical signs of illness, including changes in skin, fur, eyes, mucous membranes, salivation, and abnormal respiration, were monitored daily in both the acute oral administration study and the 28-day repeat-dose oral administration study. Abnormal neurobehaviors, such as altered gait or posture, response to handling, tonic–clonic movements, stereotypies (e.g., excessive grooming, repetitive circling), and bizarre behaviors (e.g., walking backward), were also observed.

### 4.5. Indirect Blood Pressure Measurement

Blood pressure parameters, including systolic blood pressure, diastolic blood pressure and mean arterial pressure, along with heart rate, were measured weekly using a noninvasive tail-cuff volume pressure recording sensor technology (CODA software version 4.1, Kent Scientific., Torrington, CT, USA).

### 4.6. Biochemical Analysis

Whole blood samples were collected in clot blood collection tubes and sent to the AMS Excellence Laboratory, Faculty of Associated Medical Sciences, Khon Kaen University, Thailand, to determine serum levels of electrolytes, total protein, albumin, liver enzymes (AST, ALT, ALP), total bilirubin, blood urea nitrogen (BUN), creatinine, uric acid, cholesterol, calcium (Ca^2+^), and inorganic phosphorus (PHOS) using standard procedures as a previous report [45].

### 4.7. Histological Analysis

All animals were anesthetized with sodium thiopental (50 mg/kg) (Scott-Edill Pharmacial Ltd., Solan (H.P.), India) and exsanguinated from the abdominal aorta. A full necropsy was performed on each animal, and all gross lesions were recorded. The heart, liver, and kidneys were also collected and weighed. In the 28-day repeat-dose oral administration study, vital organs, including the heart, kidneys, and liver, were examined by histopathology. These tissues were rapidly fixed in 4% paraformaldehyde and processed as previously described [46]. Briefly, the tissues were embedded in paraffin, sectioned to 5 µm thickness, and stained with hematoxylin and eosin (H&E) (Bio-Optica Milano S. p.A., Milan, Italy). Structural alterations were observed, and images were captured using an Eclipse Ni–U upright microscope (Nikon, Tokyo, Japan).

### 4.8. Statistical Analysis

Data are expressed as the mean ± standard deviation (SD). Statistical analysis was performed separately for male and female Sprague-Dawley rats using GraphPad Prism 9.5. (San Diego, CA, USA) HLO group results were compared to those of the control group using a Student’s *t*-test, with *p* < 0.05 considered significant.

## 5. Conclusions

In conclusion, our findings demonstrated that HLO contained negligible amounts of THC. Neither acute nor sub-chronic dose of HLO at 1.5 mL/kg affected body weight, organ weights, blood pressure, or electrolyte balance. There were no structural or functional abnormalities observed in vital organs, including the heart, kidneys, and liver, between sex-matched groups after acute and sub-chronic HLO administration. These results support the safety effect of HLO treatment and the prospective application of HLO in preclinical research or clinical settings. This safety profile supports the extension of research into many domains, including dose-escalation studies and extended chronic toxicity assessments. This will strengthen the evidence base for any future clinical development of HLO.

## Figures and Tables

**Table 1:** Food and water consumption for the acute oral administration and 28-day repeat-dose oral administration study in Sprague-Dawley rats.

Parameter	Male		Female	
	Vehicle	HLO	Vehicle	HLO
Acute oral administration study				
Food consumption (g/rat/day)	22.00 ± 6.63	24.40 ± 9.94	25.60 ± 4.34	23.20 ± 6.42
Water consumption (mL/rat/day)	32.00 ± 4.47	28.00 ± 8.37	30.00 ± 10.00	30.00 ± 7.07
28-day repeat-dose oral administration study				
Food consumption (g/rat/day)	21.00 ± 4.47	24.20 ± 3.27	21.40 ± 6.19	21.20 ± 1.48
Water consumption (mL/rat/day)	26.00 ± 4.18	29.00 ± 5.48	27.00 ± 9.75	29.00 ± 4.18

Data are presented as mean ± standard deviation (n = 5/group). Statistical analysis in each sex was evaluated by the unpaired *t*-test. HLO, hemp leaf oil.

**Table 2:** Vital organs weight for the acute oral administration study and 28-day repeat-dose oral administration study in Sprague-Dawley rats at termination.

Parameter	Male		Female	
	Vehicle	HLO	Vehicle	HLO
Acute oral administration study				
HW (g)	1.43 ± 0.15	1.37 ± 0.08	0.97 ± 0.05	0.91 ± 0.05
LW (g)	15.87 ± 1.63	16.10 ± 1.11	10.81 ± 0.40	10.92 ± 0.74
KW (g)	3.20 ± 0.34	3.15 ± 0.10	2.11 ± 0.17	2.07 ± 0.10
28-day repeat-dose oral administration study				
HW (g)	1.42 ± 0.06	1.45 ± 0.05	0.99 ± 0.11	1.03 ± 0.11
LW (g)	16.06 ± 1.36	16.15 ± 0.05	11.27 ± ± 0.23	11.61 ± 1.43
KW (g)	3.24 ± 0.15	3.30 ± 0.08	2.13 ± 0.15	2.12 ± 0.15

Data are presented as mean ± standard deviation (n = 5/group). Statistical analysis in each sex was evaluated by unpaired *t*-test. HW, heart weight; KW, kidney weight; LW, liver weight; HLO, hemp leaf oil.

**Table 3:** Clinical biochemistry data for the acute oral administration study in Sprague-Dawley rats at termination.

Parameter (Laboratory Historical Control Values)	Vehicle	HLO
Male Sprague-Dawley rats		
Na^+^ (131–157 mmol/L)	139.00 ± 1.73	137.40 ± 1.67
K^+^ (3.73–11.38 mmol/L)	5.58 ± 2.87	4.70 ± 0.65
Cl^−^ (93.1–112.8 mmol/L)	101.40 ± 2.61	100.40 ± 0.89
Total protein (3.4–7.7 g/dL)	4.68 ± 0.35	4.62 ± 0.04
Albumin (2.8–4.6 g/dL)	3.82 ± 0.28	3.80 ± 0.07
AST (47–266 U/L)	172.00 ± 26.12	221.75 ± 8.54
ALT (16–161 U/L)	32.00 ± 5.79	34.80 ± 8.84
ALP (46–230 U/L)	185.20 ± 33.86	172.20 ± 11.95
total Bilirubin (0.03–0.90 mg/dL)	<0.146	<0.146
BUN (8–24 mg/dL)	17.00 ± 3.87	19.00 ± 2.92
Creatinine (0.06–0.47 mg/dL)	0.26 ± 0.05	0.26 ± 0.05
Uric acid (0.5–1.5 mg/dL)	0.85 ± 0.17	0.88 ± 0.18
Cholesterol (36–163 mg/dL)	48.00 ± 3.39	51.60 ± 2.70
Ca^2+^ (9.1–13.4 mg/dL)	9.92 ± 1.00	9.76 ± 0.22
PHOS (4.6–11.7 mg/dL)	10.65 ± 2.07	11.12 ± 1.20
Female Sprague-Dawley rats		
Na^+^ (128–159 mmol/L)	140.20 ± 0.84	140.00 ± 2.83
K^+^ (3.49–12.96 mmol/L)	4.68 ± 0.18	4.68 ± 0.61
Cl^−^ (89.1–114.6 mmol/L)	106.80 ± 3.27	105.60 ± 4.28
Total protein (5.1–9.0 g/dL)	5.32 ± 0.44	5.28 ± 0.19
Albumin (2.6–6.4 g/dL)	4.68 ± 0.36	4.66 ± 0.13
AST (42–341 U/L)	76.20 ± 9.65	90.60 ± 13.18
ALT (13–182 U/L)	19.80 ± 2.28	22.20 ± 5.17
ALP (15–115 U/L)	105.4 ± 33.81	108.8 ± 22.29
total Bilirubin (0.04–0.25 mg/dL)	<0.146	<0.146
BUN (8–28 mg/dL)	18.40 ± 4.34	18.40 ± 2.30
Creatinine (0.11–0.53 mg/dL)	0.28 ± 0.08	0.32 ± 0.04
Uric acid (0.5–1.5 mg/dL)	0.82 ± 0.24	0.82 ± 0.29
Cholesterol (28–249 mg/dL)	54.00 ± 5.34	56.60 ± 8.88
Ca^2+^ (7.7–15.5 mg/dL)	9.88 ± 0.65	9.88 ± 0.44
PHOS (2.4–12.4 mg/dL)	11.04 ± 0.54	10.92 ± 2.12

Data are presented as mean ± standard deviation (n = 5/group). ALP, alkaline phosphatase; ALT, alanine aminotransferase; AST, aspartate aminotransferase; BUN, urea nitrogen; Ca^2+^, calcium; Cl^−^, chloride; K^+^, potassium; Na^+^, sodium; PHOS = inorganic phosphorus; HLO, hemp leaf oil.

**Table 4:** Clinical biochemistry data for the 28-day repeat-dose oral administration study in Sprague-Dawley rats at termination.

Parameter (Laboratory Historical Control Values)	Vehicle	HLO
Male Sprague-Dawley rats		
Na^+^ (131–157 mmol/L)	138.60 ± 0.55	137.60 ± 0.89
K^+^ (3.73–11.38 mmol/L)	4.42 ± 0.27	4.74 ± 0.40
Cl^−^ (93.1–112.8 mmol/L)	104.8 ± 1.30	104.2 ± 1.79
Total protein (3.4–7.7 g/dL)	4.44 ± 0.17	4.48 ± 0.18
Albumin (2.8–4.6 g/dL)	3.70 ± 0.14	3.74 ± 0.21
AST (47–266 U/L)	194.20 ± 16.75	173.60 ± 11.70
ALT (16–161 U/L)	25.60 ± 3.44	21.20 ± 2.86
ALP (46–230 U/L)	118.20 ± 18.66	113.80 ± 16.30
total Bilirubin (0.03–0.90 mg/dL)	<0.146	<0.146
BUN (8–24 mg/dL)	15.60 ± 2.19	18.40 ± 2.61
Creatinine (0.06–0.47 mg/dL)	0.28 ± 0.08	0.32 ± 0.04
Uric acid (0.5–1.5 mg/dL)	1.12 ± 0.16	1.14 ± 0.27
Cholesterol (36–163 mg/dL)	51.60 ± 6.43	46.40 ± 7.70
Ca^2+^ (9.1–13.4 mg/dL)	9.82 ± 0.29	9.70 ± 0.35
PHOS (4.6–11.7 mg/dL)	9.94 ± 0.61	9.06 ± 0.59
Testosterone (100–300 ng/dL)	341.60 ± 90.45	272.75 ± 130.34
Female Sprague-Dawley rats		
Na^+^ (128–159 mmol/L)	138.20 ± 2.49	137.20 ± 2.59
K^+^ (3.49–12.96 mmol/L)	4.60 ± 0.85	4.82 ± 1.21
Cl^−^ (89.1–114.6 mmol/L)	108.80 ± 0.45	104.60 ± 3.85
Total protein (5.1–9.0 g/dL)	5.70 ± 0.22	5.66 ± 0.55
Albumin (2.6–6.4 g/dL)	5.05 ± 0.17	5.10 ± 0.50
AST (42–341 U/L)	77.50 ± 10.47	89.25 ± 11.00
ALT (13–182 U/L)	28.60 ± 14.10	24.00 ± 3.39
ALP (15–115 U/L)	103.40 ± 23.02	71.20 ± 14.27
total Bilirubin (0.04–0.25 mg/dL)	<0.146	<0.146
BUN (8–28 mg/dL)	18.60 ± 1.82	21.80 ± 3.27
Creatinine (0.11–0.53 mg/dL)	0.30 ± 0.00	0.34 ± 0.05
Uric acid (0.5–1.5 mg/dL)	1.20 ± 0.22	1.08 ± 0.28
Cholesterol (28–249 mg/dL)	60.20 ± 4.15	59.80 ± 6.71
Ca^2+^ (7.7–15.5 mg/dL)	10.28 ± 0.50	10.66 ± 1.24
PHOS (2.4–12.4 mg/dL)	8.15 ± 0.83	7.54 ± 0.75
Estrogen (10–50 pg/mL)	27.75 ± 11.84	41.20 ± 18.03

Data are presented as mean ± standard deviation (n = 5/group). ALP, alkaline phosphatase; ALT, alanine aminotransferase; AST, aspartate aminotransferase; BUN, urea nitrogen; Ca^2+^, calcium; Cl^−^, chloride; K^+^, potassium; Na^+^, sodium; PHOS = inorganic phosphorus; HLO, hemp leaf oil.

**Table 5:** Specifications of the hemp leaf oil (HLO).

Parameter	Specification	Testing Method
Identification		
Aroma and visual	Olive oil aroma	olfactory
	Clear, amber color	visual
	Free for foreign material	visual
pH	5.88	In-house method based on AOAC (2019) 943.02 [37]
Phytochemicals		
CBD	0.40 mg/g	HPLC
THC	0.02 mg/g	HPLC
Microbiology		
Coliforms	2.3 MPN/mL	FDA BAM 2017 (chapter4) [38]
*Escherichia coli*	<0.3 MPN/mL	FDA BAM 2017 (chapter 4) [38]
*Salmonella* spp.	Not detected	ISO 6579-1:2017 [39]
*Staphylococcus aureus*	Not detected	FDA BAM 2016 (chapter 12) [40]
Total aflatoxin	Not detected	In-house method TE-CH-025 based on AOAC (2016) 991.31 and 994.08 [41]
Heavy metals		
Arsenic (As)	Not detected	Analyst, August 1994 [42]
Cadmium (Cd)	Not detected	Analyst, August 1994 [42]
Lead (Pb)	Not detected	Analyst, August 1994 [42]
Mercury (Hg)	Not detected	Analyst, August 1994 [42]
Tin (Sn)	2.453 mg/kg	In-house method based on AOAC (2000) 985.16 [43]

BAM, Bacteriological Analytical Manual; CBD, cannabidiol; FDA, Food and Drug Administration; HPLC, High Performance Liquid Chromatography; THC, tetrahydrocannabinol.

## Data Availability

Data presented in this study is contained within the article. Further inquiries can be directed to the corresponding author.

## Abbreviations

The following abbreviations are used in this manuscript:

HLO	Hemp leaf oil
THC	Tetrahydrocannabinol
CBD	Cannabidiol
BAM	Bacteriological Analytical Manual
FDA	Food and Drug Administration
HPLC	High Performance Liquid Chromatography
